# The Therapeutic Application of Chloroform in Labor*Read before the Richmond Academy of Medicine and Surgery, March 9, 1897.

**Published:** 1897-04

**Authors:** John N. Upshur

**Affiliations:** Richmond, Va., Professor of Practice of Medicine in Medical College of Virginia, President of the Richmond Academy of Medicine and Surgery, etc.


### THE THERAPEUTIC APPLICATION OF CHLOROFORM IN LABOR.*

By JOHN N. UPSHUR, M.D., Richmond, Va.,

Professor of Practice of Medicine in Medical College of Virginia, President of the Richmond Academy of Medicine and Surgery, etc.

Since the primeval curse fell upon our race, and pain and anguish have been the invariable and dreaded accompaniment of man's entry into the world, to soothe woman's sorrows and conduct her safely through the crisis is an object worthy our highest effort, and one to be sought for with commendable devotion.

To accomplish this end the administration of chloroform has become routine practice, and the consensus of opinion, from a very large number of obstetricians, is in favor of its safety when thus exhibited. Careful observation for many years has tended to make me question its utility in many cases, nay, to convince me that oftentimes it *adds* to the peril and prolongs the trials.

That loyal devotion to true obstetric science demands that chloroform in labor should be exhibited just *as other therapeutic* agents in the treatment of other maladies. We forget that delivery is not a pathological process, but a physiological function; that it becomes pathological only when conditions arise which convert a eutocia into a dystocia, or when the physical conformation of the pelvis is such as to prevent the accomplishment of natural delivery by mechanical obstruction, from errors in the pelvic conformation, malposition of the child or monstrosity. When labor has become pathological in character and operative interference, manual or instrumental, is demanded, with the dangers from exhaustion from prolonged agony or the addition of surgical shock, the case passes to the domain of the surgeon, so far as the *unquestioned exhibition* of chloroform is concerned, just as an anesthetic is demanded in any surgical procedure as a safeguard to life. Or when the condition of the mother is such that her life is imperiled by the super-

*Read before the Richmond Academy of Medicine and Surgery, March 9, 1897.

vention of convulsions from existence of any of the systemic conditions which, predisposing to such complication, become active factors so soon as the stimulus of pain, or the concentration of poison to the nervous system sets in motion the morbid phenomena, which manifest themselves by an outward explosion, jeopardizing the life of the infant and of the mother.

It is not with conditions such as these that I purpose dealing, but with chloroform therapeutically applied as a *causative* factor, either predisposing or active, in transforming this physiological function into a condition of some form of dystocia, making aid or interference necessary to the safe delivery of the woman.

The question, therefore, resolves itself into this problem: in what cases should chloroform be administered? at what stage of labor? what dangers arise in consequence? at what stage do these complications arise; the best means of combating them? and, finally, *is it justifiable* to administer chloroform in natural labor *progressing with satisfactory rapidity?*

To satisfactorily deal with these queries, we must discuss the physiological action of chloroform so far as it bears upon this question. In the first stage of the *administration* of chloroform, we have its stimulant effect. The second is the stage of narcosis. In the third stage, the functions of the spinal chord are abolished, as are those of the brain—this is the surgical stage; and in the fourth, we have the condition of complete paralysis—death, usually by overpowering the respiratory center first. The modes of death are from reflex irritation of the cardiac ganglia; from epileptiform syncope in the stage of stimulation; from paralysis of respiration; from paralysis of the heart; and, finally, from depression by the chloroform narcosis and shock. Chloroform *diminishes* the *excitability* of the *muscular system* and *its capacity for work.* If prolonged, it *exhausts muscular irritability.* It interferes with *oxidation of the blood,* and thus becomes *toxic to the fetus.* Its stimulant action on the vaso-motor center is doubtful.

Now, as a practical deduction from these *facts* as to the action of chloroform, let us apply them to the solution of the queries as enunciated above. As to suitable cases for the administration, we need not consume time in discussing; every skillful and experienced obstetrician will point to those cases in which the pains are nagging and exhausting—in rigid os, in great nervousness and restlessness. I think, too, that except, as just stated, it should not be administered until the *second stage* of *labor* is *well established,* and at the *latter part* of that stage, and *withdrawn* so soon as the occiput has passed the ostium vaginæ.

The most serious question is as to the danger arising from the exhibition of chloroform in labor, and it is with this question I would deal with the greatest emphasis. Note the danger from *reflex* irritability, with incomplete anesthesia; it is at least worthy of investigation that if fatal influence from chloroform does not manifest itself here, still its effect on the heart, through the cardiac ganglia, may be potent enough to at least predispose to hemorrhage, or to weakened heart action during convalescence, with the condition of debility consequent upon it. But the obstetric stage is the beginning of *narcosis;* it is difficult to keep the patient at this point. Lack of appreciation of what is going on interferes with voluntary assistance. One of its physiological effects is to diminish muscular excitability, to interfere with muscular capacity for work. Pains are consequently less potent and effective in expelling the child; there is much greater muscular tire, and the natural consequence is that when the second stage is completed, danger of hemorrhage is much greater from incomplete condensation of the uterus, and I have often seen it occur. The uterus is left in such condition as to make *subinvolution,* with all the *ills* that *follow in its train, almost inevitable.*

The spongy condition of the womb makes the patient much more liable to septic infection. Not only so, but often have I seen labor suspended; nor on the withdrawal of the chloroform, did the uterine pains return with desirable efficiency; evidently its effect on the uterine muscle was toxic in character, and instrumental delivery became a necessity, the agent having transformed a simple physiological process into a pathological one,—thus adding to the peril of the mother. The interference with oxidation of the blood endangers the life of the child, and I make bold to assert my belief that its use in natural labor *increases the percentage of still-births.* Nor are these the most serious risks. Though few deaths have been reported from chloroform in obstetric practice, it is a question if death supervening in many cases within forty-eight hours after delivery, and reported as heart-clot, etc., may not have been due to the depression from long-continued administration of chloroform, plus the shock of labor. In the cases where uterine contraction is not interfered with, and the woman holds her breath, fixing the chest-wall to more efficiently bear down, she is put in a most favorable condition for the occurrence of epileptiform syncope, when chloroform is being administered; and the retention of carbonized blood in the brain may under such conditions overwhelm the nervous system. The dangers from chloroform exhibition are most likely to manifest themselves, however, at the close of the second, or during the third stage of labor, and here post-partum, or accidental hemorrhage, is the most important, and may be the focus from which may radiate other serious evils.

I would lay down the proposition as an axiom, that *whenever* chloroform has been administered, a full dose of ergot should be given so soon as the head is delivered or the second stage of labor completed. I have never regretted giving the ergot, and remember *no case* in which it is omitted that I did not repent of the delinquency. I would also suggest that it is safe practice to give a full dose of quinine (gr. x) at the beginning of the second stage of labor when chloroform is to be exhibited, or belladonna, or one or more doses of nitroglycerine; especially will this last tend to antidote the nausea following chloroform administration.

The hypodermic injection of atropine (gr. $\frac{1}{120}$), or sulphate of strychnia (gr. $\frac{1}{60}$), will undoubtedly add to the safety of the patient.

We must not lose sight of the fact that in obstetric use of chloroform (which is usually conceded as the safest field for the use of chloroform), the patient is *much longer* under the influence of the anesthetic, and that the stimulus of constantly recurring uterine contractions, of a painful character, *which are supposed to antidote* the ill effects of the chloroform by constantly arousing the patient, may be absent, or fail to exercise the stimulant effect usually a consequence of pain.

In the light of the foregoing facts, I most earnestly avow my belief that we, as physicians, should place chloroform upon the same platform as other drugs; not be influenced by our sympathies aroused by the pleadings of patients, or the fashionable routine practice of the day, but initiate and sustain a much needed reform in our obstetric work, chloroform being administered, as other agents, when the indications in the cases imperatively demand it—not unless. He is a bold man who, invading the domain of nature, interferes with her physical processes, and places the wife and mother in a position of increased peril, and perchance, the shadow of a fatal issue, or, at least, a life of invalidism and suffering, where, before, the home was irradiated with the sunlight of true and unalloyed happiness.

*210 West Grace Street.*

